# Dual properties of a hydrogen oxidation Ni-catalyst entrapped within a polymer promote self-defense against oxygen

**DOI:** 10.1038/s41467-018-03011-7

**Published:** 2018-02-28

**Authors:** Alaa A. Oughli, Adrian Ruff, Nilusha Priyadarshani Boralugodage, Patricia Rodríguez-Maciá, Nicolas Plumeré, Wolfgang Lubitz, Wendy J. Shaw, Wolfgang Schuhmann, Olaf Rüdiger

**Affiliations:** 1 Max-Planck-Institut for Chemical Energy Conversion, Stiftstrasse 34–36, 45470 Mülheim an der Ruhr, Germany; 2 Department Analytical Chemistry, Center for Electrochemical Sciences (CES), Ruhr-Universität Bochum, Universitätsstrasse 150, 44780 Bochum, Germany; 3 Pacific Northwest National Laboratory, 902 Battelle Blvd, Richland, WA 99352 USA; 4 Center for Electrochemical Sciences—Molecular Nanostructures, Ruhr-Universität Bochum, Universitätsstrasse 150, 44780 Bochum, Germany

## Abstract

The Ni(P_2_N_2_)_2_ catalysts are among the most efficient non-noble-metal based molecular catalysts for H_2_ cycling. However, these catalysts are O_2_ sensitive and lack long term stability under operating conditions. Here, we show that in a redox silent polymer matrix the catalyst is dispersed into two functionally different reaction layers. Close to the electrode surface is the “active” layer where the catalyst oxidizes H_2_ and exchanges electrons with the electrode generating a current. At the outer film boundary, insulation of the catalyst from the electrode forms a “protection” layer in which H_2_ is used by the catalyst to convert O_2_ to H_2_O, thereby providing the “active” layer with a barrier against O_2_. This simple but efficient polymer-based electrode design solves one of the biggest limitations of these otherwise very efficient catalysts enhancing its stability for catalytic H_2_ oxidation as well as O_2_ tolerance.

## Abstract (web summary)

Bio-inspired Ni-based molecular catalysts are efficient for H_2_ oxidation, but are suffering from the poor stability in the presence of O_2_. Here, the authors develop a strategy to boost greatly their stability by dispersing them in a hydrophobic and redox-silent polymer matrix.

## Introduction

A major challenge of humankind is a future sustainable energy economy. Hydrogen has been proposed as an ideal target to store energy from renewable sources, e.g., solar-driven water splitting^1–4^. A hydrogen-powered fuel cell is then able to recover a major part of the energy in high yields on demand. An unsolved challenge in this endeavor is to design active, efficient and stable catalysts based on earth-abundant metals^4^.

Nature uses the highly active and efficient hydrogenase enzymes for hydrogen cycling in living systems. The enzymes are capable of achieving low overpotentials and high turnover frequencies in both hydrogen oxidation and proton reduction, bearing only the abundant metals Fe and/or Ni in their active sites^5^. Chemists have been inspired by these enzymes to design inexpensive molecular complexes capable of efficiently producing or oxidizing H_2_. Particularly noteworthy examples are the DuBois catalysts^6,7^, which are Ni-(bis)diphosphine based complexes equipped with a pendant amine that acts as a Lewis base, accepting the proton during H_2_ splitting, in a manner analogous to that proposed to occur at the active site of [FeFe] hydrogenases^8,9^.

Extension of the proton channel with a carboxylic acid moiety between the metal center and the solvent by attachment of an amino acid to the pendant amine, allows the DuBois catalyst to operate at very low overpotentials at low pH and at room temperature in aqueous systems^10–12^, or even reversibly^13,14^. Moreover, these catalysts show tolerance towards CO, a common contaminant of H_2_ feedstocks and a strong inhibitor of hydrogenases and of platinum used for catalysis^5,15,16^.

**Fig. 1:**
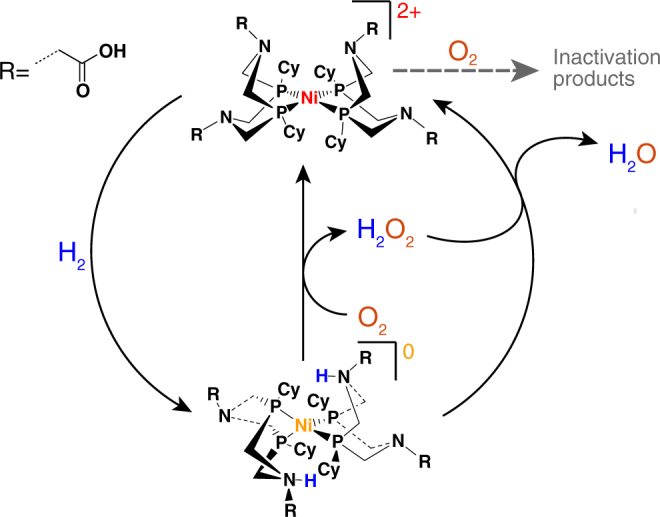
Reactions of the CyGly complex with H_2_/O_2_ in solution. The Ni^2+^ complex, [Ni^II^(P^Cy^_2_N^Gly^_2_)_2_]^2+^, undergoes slow and irreversible inactivation by O_2_ while the H_2_ reduced Ni^0^ complex, [Ni^0^(P^Cy^_2_N^Gly^_2_)_2_], is able to catalytically reduce O_2_ to H_2_O

On the other hand, the DuBois catalyst undergoes rapid degradation under conditions relevant for technological applications, i.e., when immobilized on an electrode^15,17,18^ or on photoactive materials^19^. We demonstrated in a previous study that the electrocatalytic H_2_ oxidation activity of the DuBois catalyst CyGly (for Structure see Fig. 1) was lost irreversibly after 10 min when 2% O_2_ was added to the H_2_ gas feed^15^. Similarly, other studies with DuBois complexes in solution revealed that the H_2_ production activity was completely suppressed when O_2_ was present in the electrochemical cell^16,20^. Crystallographic and nuclear magnetic resonance (NMR) spectroscopic studies showed that the Ni-(bis)diphosphine complexes in low oxidation states react with O_2_ to oxidize the phosphine ligands, inactivating the complexes irreversibly^21^. Hence, under electrocatalytic conditions implying fast collection of electrons by the electrode, which is desired for current generation in fuel cells, exacerbates the O_2_ sensitivity of the Ni-catalyst. In contrast, in solution and in the presence of H_2_, the Ni-complex accumulates in a highly reduced doubly protonated Ni^0^ state, which can catalytically reduce O_2_ to water, slowing the formation of the oxidized inactive complex (Fig. 1)^21^.

In the present study, we take advantage of the dual catalytic nature of this complex: the electrocatalytic oxidation of H_2_ and the catalytic reduction of O_2_. We use here the properties of a redox-silent hydrophobic polymer to support the catalyst and obtain a polymer/catalyst film which creates two discrete reaction layers serving separate roles: a phase close to the electrode surface where electrons can directly tunnel between catalyst and electrode. This layer is responsible for electrocatalytic H_2_ oxidation. Near the interface between the polymer and the electrolyte, a second region which is electrically disconnected from the electrode surface, prevents anodic reoxidation of the catalyst, allowing the doubly protonated Ni^0^ complex to reduce incoming O_2_ to water. This results in a simple and efficient self-protecting, catalytically active matrix that serves to extend the limited lifetime of the Ni-catalyst when immobilized on an electrode surface.

## Results

### Reactivity of the CyGly complex on electrode surfaces with O_2_

**Fig. 2:**
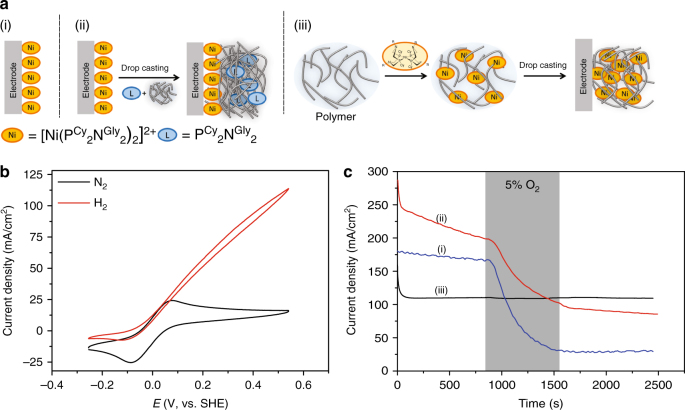
Catalytic properties and tolerance of CyGly modified electrodes towards oxygen. **a** Electrode preparation schemes: (i) A monolayer of CyGly complex; (ii) a monolayer of CyGly complex coated with the pristine polymer and the P^Cy^_2_N^Gly^_2_ ligand, and (iii) CyGly/polymer film on glassy carbon electrodes. **b** Cyclic voltammograms of the polymer/catalyst film (iii) under N_2_ (black trace) and under H_2_ (red trace). Conditions: 20 mV s^−1^, pH = 3, 0.1 M MES/0.1 M HEPES + HClO_4_, 25 °C, 2000 rpm. **c** Chronoamperometry experiments under 90% H_2_/10% N_2_ with the addition of 5% O_2_ (90% H_2_, 5% N_2_) over 600 s (shaded area) for all three electrode preparations. Conditions: + 541 mV vs. SHE, 25 °C, 2000 rpm, pH = 3, 0.1 M MES/0.1 M HEPES + HClO_4_. The initial current drop corresponds to slow equilibration processes inside the polymer (establishment of H_2_ and Ni^2+^ gradients)

The catalyst was dispersed into a poly(glycidyl methacrylate-*co*-butyl acrylate-*co*-poly(ethylene glycol) methacrylate) (denoted as P(GMA-BA-PEGMA) in the following) polymer (See Methods section)). This polymer was chosen because of its rather hydrophobic nature that allows for the formation of stable films in aqueous electrolytes. Moreover, the polymer backbone consists of chemically inert and non-coordinating monomers. Thus, unintended interactions between the Ni-center and the polymer backbone that may affect the catalytic properties of the catalyst are prevented. Film formation was carried out by drop-casting of a homogenous solution of the polymer and the complex in acetonitrile on a glassy carbon electrode followed by drying under anaerobic conditions. Consecutive cyclic voltammograms of the modified electrodes at pH 3.0 revealed the appearance of a reversible signal with a mid-point potential of −12 mV vs. SHE (Fig. 2). This wave was previously assigned to two overlapping one electron processes, corresponding to the reduction of Ni^2+^ to Ni^0 ^^10,15^. The intensity of the signal increases during the first 30 min as a result of the initial solvation of the polymer and/or penetration of counter ions into the film (see Supplementary Fig. 1). The peak intensity is proportional to the square root of the scan rate, indicating the diffusional nature of the electrochemical process. This is indicative of some mobility of the Ni-complex, and/or counter ion transport inside the polymer film (See Supplementary Fig. 2). When H_2_ is flushed through the electrochemical cell, a catalytic current appears starting at the redox potential of the complex (Fig. 2b). The catalytic current increases with the applied potential, indicating slow counter ion movement within the film or a distribution of the tunneling distance between the CyGly molecules and the electrode as a result of the non-conductive nature of the polymer. The latter is similar to what has been reported for complexes in solution with bulky dipeptides in the outer coordination sphere^22^.

Addition of O_2_ to the gas mixture during H_2_ oxidation using a film of CyGly complex dispersed in the polymer, does not affect the catalytic current (Fig. 2c, black trace). This is in stark contrast to measurements with a monolayer of the immobilized catalyst, where the CyGly complex loses 75% of its catalytic H_2_ oxidation activity irreversibly over 10 min as 5% O_2_ is added to the gas feed (Fig. 2c, blue trace). On the other hand, when the catalyst in a DET configuration is exposed to O_2_ in the absence of H_2_, most of the catalytic activity is recovered when the gas is switched back to H_2_ (See Supplementary Fig. 3b, blue trace). This suggests a higher O_2_ sensitivity of the complex during electrocatalytic H_2_ oxidation. When the potential on the electrode was kept low enough to maintain the Ni-center in the reduced Ni^0^ state during O_2_ exposure, the catalytic current was completely recovered (see Supplementary Fig. 3b, red trace). In line with this result, electrodes prepared outside the glovebox did not show any catalytic activity (data not shown) suggesting that long exposure of the Ni-complex in the Ni^2+^ oxidation state to O_2_ without any applied potential on the electrode or in the absence of H_2_ damages the catalytic properties of the complex.

To evaluate if the observed protection is only based on a simple physical blocking of O_2_ by the polymer, cyclic voltammetry experiments with polymer-coated glassy carbon electrodes in the presence of O_2_ were conducted. Indeed, direct O_2_ reduction at glassy carbon electrodes is significantly hampered by the presence of the polymer film on the electrode surface (See Supplementary Fig. 4). However, when a monolayer of the catalyst on the electrode surface was further coated with a mixture of polymer and ligand (of similar thickness, with two equivalents of CyGly ligand but without Ni), the catalyst exhibits similar sensitivity to oxygen as the monolayer without a polymer top layer (Fig. 2c, red trace). This excludes on one hand the possibility that a physical barrier to oxygen is the sole explanation for the decreased oxygen sensitivity in catalyst-polymer films. On the other hand, since the CyGly ligand is also O_2_ sensitive, this experiment further demonstrates that the simple stoichiometric reaction of O_2_ with the catalyst during its oxidative degradation does not significantly participate in the protection mechanism. An active Ni-complex dispersed in the polymer film is required for effective protection.

### The CyGly complex as an O_2_ reducing catalyst

The reactivity of the CyGly complex with O_2_ in solution was analyzed by means of ^2^H NMR spectroscopy. D_2_ was used as a reductant and the formation of D_2_O upon O_2_ addition to the gas feed was quantified. The TOF for O_2_ reduction to D_2_O was measured to be (20 ± 5) h^−1^ (see Supplementary Fig. 5). Formation of D_2_O under such conditions supports the mechanism proposed in Fig. 1 and is consistent with results described previously by Yang et al^21^. for a series of similar complexes containing a pendant base on the phosphine ligand (see Supplementary Note 1).

On the other hand, if the NMR tube contains mixtures of H_2_ and O_2_ in order to reproduce the conditions in the electrochemical cell, the ^31^P NMR spectrum of the catalyst in solution shows almost complete loss of the CyGly resonances after 3 h along with new resonances of its degradation products appearing (See Supplementary Figs 11 and 12).

To reduce O_2_ to water, two molecules of the Ni^0^ complex are needed to provide the required 4 electrons and protons (Fig. 1). This is possible with the freely diffusing catalyst in solution, but using a monolayer of catalyst immobilized on the electrode, the catalyst is cycling through different redox states and geometries under turnover conditions^23^. As a result, the immobilized doubly protonated Ni^0^ complex does not accumulate in the required concentrations to effectively reduce O_2_ to water, because of fast electron transfer to the electrode. Consequently, catalyst degradation and the observed drop in catalytic current results (Fig. 2c, red and blue traces).

### A polymer matrix to separate reaction layers

**Fig. 3:**
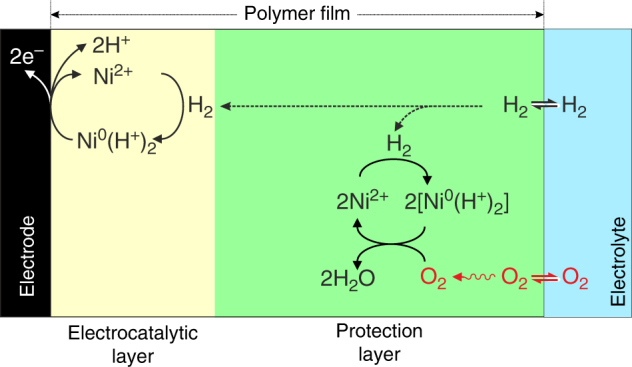
Proposed reactions inside the polymer/catalyst film. Schematic representation of the polymer film separating the immobilized CyGly complex into two functional layers where different reactions occur; colors are used to identify the different polymer layers and the boundary between polymer and electrolyte. In the electrocatalytic layer (yellow), H_2_ is oxidized to protons and electrons via the Ni-complex. Within the protection layer (green) the doubly reduced Ni^0^ complex catalyzes the reduction of incoming O_2_ to H_2_O by using the electrons supplied from H_2_

The stability of the H_2_ oxidation currents under O_2_ observed in Fig. 2c for the CyGly/polymer films can be explained if we consider a layered structure for the CyGly complex/polymer film. The insulating polymer acts as a supporting matrix to immobilize the complex on the electrode surface, but also prevents electrical contact between the complexes in the remote regions of the film and the electrode. The formation of a sufficiently thick polymer film generates two distinct regions; with electrocatalytic H_2_ oxidation in the inner layer near the electrode surface and O_2_ reduction in the outer layer as shown in Fig. 3.

Under H_2_ in the absence of O_2_, the complex in the protection layer (Fig. 3, green region) is reduced to the protonated Ni^0^ state, but since it is electrically isolated from the electrode, electrons will not be shuttled to the electrode. However, when O_2_ reaches the film/electrolyte interface, it will encounter the reduced Ni-complexes, which will then reduce incoming O_2_ to water, thus protecting the electrocatalytic layer (Fig. 3, yellow region) from O_2_ damage.

### Validation of the protection mechanism

The mechanism of O_2_ reduction to water requires two catalyst molecules per O_2_, which has implications for the film preparation. If films with low catalyst loading are used, oxidative damage of the catalyst is observed (See Supplementary Fig. 6). This is thought to be due to slow diffusion of the catalyst molecules within the film, thus allowing O_2_ to pass the protection layer and/or the accumulation of partially reduced oxygen species.

**Fig. 4:**
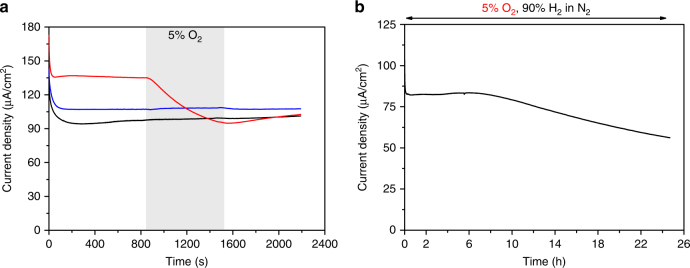
Catalytic H_2_ oxidation by CyGly/polymer films in the presence of O_2_. **a** Chronoamperometric experiments of glassy carbon electrodes (GCEs) modified with CyGly/polymer films of different thicknesses obtained by drop-casting 20 µL (black line), 10 µL (blue line) and 2.5 µL (red line) of the CyGly/polymer solution in acetonitrile onto the GCEs. Measurements conducted under 90% H_2_/10% N_2_ with addition of 5% O_2_ (90% H_2_/5% N_2_) over 10 min (shaded area). Conditions: + 541 mV vs. SHE, pH = 3, 0.1 MES/0.1 M HEPES + HClO_4_, 25 °C, 500 rpm. **b** Chronoamperometric experiment with a CyGly/polymer film under 5% O_2_/90% H_2_/5% N_2_ gas mixture over 24 h showing the high stability of the H_2_ oxidation process in this film even under aerobic conditions. Conditions: + 541 mV vs. SHE, pH = 3, 0.1 M MES/0.1 M HEPES + HClO_4_, 25 °C, 2000 rpm

Conductive multi-walled carbon nanotubes (MWCNT) were used to extend the electrocatalytic region in the polymer film, connecting the electrode to catalyst molecules in the film further away from the electrode, i.e., in the previously defined protection layer. The resulting electrode showed higher H_2_ oxidation currents, but as expected, reduced the protection layer by increasing the number of catalyst molecules capable of H_2_ electrocatalysis rather than O_2_ reduction. Consequently, the result is a catalytic current decay when O_2_ is added to the gas feed (see Supplementary Fig. 7). Moreover, the thickness of the film is crucial for effective protection. While thinner films produced higher catalytic currents for H_2_ oxidation (substrate diffusion is not limiting), these films do not show protection against O_2_ and the H_2_ oxidation current decayed as soon as O_2_ was added to the gas feed (Fig. 4a). Similar O_2_ sensitivity as a function of film thickness was observed for an air sensitive enzyme, i.e., a NiFeSe hydrogenase, that was immobilized in an O_2_ quenching redox polymer^24^.

To further investigate the catalytic nature of the O_2_ reduction reaction by the catalyst, we tested the stability of the catalytic current when the film was exposed to O_2_ in the absence of H_2_. This gas is required to regenerate the reduced Ni-complex in the protection layer once it is oxidized by an O_2_ molecule. Supplementary Fig. 8 shows that when the catalyst-polymer film is exposed to 5% O_2_ in the absence of H_2_ for 5 h, 35% of the catalytic activity is lost, while when H_2_ is present, 100% of the initial current is maintained for the same O_2_ exposure. Interestingly, while measuring the control experiment under N_2_ (See Supplementary Fig. 8, blue trace), without O_2_ to corroborate that N_2_ is not damaging the complex, we noticed that the oxidation current did not reach 0 A. The most likely explanation for this observation is that as we start the experiment under H_2_ to record the initial activity of the film, the catalyst in the polymer layer is completely reduced by H_2_. Since diffusion inside the polymer is extremely slow, its oxidation by the electrode surface is also very slow. Integration of the charge passed gives a value of 31 μC, which would correspond to 0.2 μmol of Ni-complex, 20% of the catalyst present in the film. This explains why only 35% of the catalytic activity is lost under a N_2_/O_2_ mixture. Under such conditions, the Ni-complex in the polymer film is still reduced, and therefore protecting the complex in the inner layer, but in the absence of H_2_ to regenerate the reduced complex, the inactive front advances more rapidly.

### The polymer enhances catalyst stability

The catalytic current was remarkably stable over time. After 18 days of continuous measurement in a chronoamperometry experiment under turnover conditions in the absence of O_2_, the electrode still maintained 75% of the initial current (See Supplementary Fig. 9). This stability contrasts with what was reported previously for a monolayer of the same catalyst covalently attached to the electrode, where the activity was completely lost after 3 days^15^. The increased stability could be a result of the hydrophobic nature of the polymer film, which may stabilize the integrity of the complex during turnover. Moreover, within the polymer film, the Ni-catalyst can freely diffuse without any structural and/or conformational constraints, as it is the case for the surface confined Ni-catalyst (See Supplementary Note 2).

The H_2_ oxidation current remains completely unaffected by the presence of O_2_ in the gas feed for the first 7 hours (Fig. 4b). After this time, the current starts to decay slowly, maintaining 75% of the initial current after 24 h of continuous O_2_ exposure. As stated before, we cannot eliminate the undesired slow oxidation of the phosphines^21^ forming an inactive product and therefore eliminating the protective capacity of the protection layer over time. After 7 h the catalyst in the protection layer is most likely starting to get oxidized, which results in an advancement of the inactive front reaching the catalyst molecules in the electrocatalytic layer. At this point, catalyst molecules participating in the electrocatalytic H_2_ oxidation begin to decompose due to incoming O_2_, resulting in the slow decay of the catalytic current.

## Discussion

DuBois catalysts with modifications in the outer coordination sphere have already been proposed as good candidates for use in fuel cells^10,14,23,25,26^. They operate at very low overpotential and unlike noble metals or hydrogenases they are insensitive to CO,^16,17^ which allows for the use of inexpensive low purity H_2_ as fuel. The remaining limitations to be solved are the lack of stability and the oxygen sensitivity^16^. We demonstrate that the use of a redox silent polymer acting as a support for the fabrication of H_2_ oxidation may overcome these limitations. The polymer matrix provided several important advantages to the Ni-complex: the stability of the catalyst was extended dramatically, both in the absence and in the presence of O_2_. While previous strategies for protection of enzymes against oxidative damage were based on redox polymers with intrinsic O_2_ reducing capabilities^24,27–29^, here the polymer is redox silent and does not contribute directly to protection via a redox process. Instead, the polymer serves as a supporting matrix that imposes local redox states and confers local reactivity to the embedded catalysts. For sufficiently thick films, the polymer electrically isolates the catalyst in the outer layer (protection layer) from the electrode. As a result, when exposed to H_2_, this portion of the catalyst remains in the Ni^0^ state and is capable of reducing incoming O_2_. This allows the catalyst to effectively eliminate O_2_ in the outer layers of the polymer, preventing it from reaching the electrocatalytic H_2_ oxidation layer. Moreover, it is possible to achieve high catalyst concentrations inside the polymer film to favor fast interaction of O_2_ with two reduced Ni-complexes to ensure the complete reduction of O_2_ to water. It is the combination of these effects that makes the O_2_ protection mechanism possible.

This possibility to activate the catalyst for self-protection without the need for auxiliary functionalities greatly simplifies the protection concept. Importantly, the ability to maintain the metal complex in a highly reduced protonated state, which is essential for protection, scales with the activity for H_2_ oxidation, implying that future highly active catalysts, even if potentially highly oxygen sensitive, may still find technological application by exploiting this protection concept.

## Methods

### Electrochemical experiments

All electrochemical experiments, electrode modifications and handling of the CyGly were carried out inside a glovebox (MBRAUN) filled with nitrogen. A set of mass flow-controllers (Brooks Instruments) were used to control the gas composition flushed through the electrochemical cell. The total flow in all experiments was 1000 mL min^−1^, unless stated otherwise. An oxygen filter (Air Liquide) before the electrochemical cell avoids any undesired O_2_ contamination. The potential was controlled by a VersaSTAT 4-400 potentiostat. A standard three electrode water jacketed electrochemical cell was used for the measurements with a Pt wire as counter electrode and a saturated calomel electrode located in a side arm as reference electrode. All potentials were converted to the standard hydrogen electrode (SHE) by adding + 241 mV.

### NMR experiments

A DPX200 NMR spectrometer from Bruker with a proton resonance frequency of 200.13 MHz was used for polymer characterization and a Bruker Avance500 (202.4 MHz ^31^P resonance frequency) for ^31^P NMR measurements. Solution state ^2^H NMR spectra were recorded on an Agilent VNMR spectrometer (500 MHz ^1^H resonance frequency). Direct detect dual-band or OneNMR probes were used. Typical ^2^H 90^o^ pulses were ∼10 μs. The polymer was characterized in deuterated acetone-d6. The residual solvent peak was used as the internal standard.

### Ni complex and polymer synthesis

The synthesis of the CyGly complex was described earlier^10^. All chemicals and solvents for polymer synthesis were purchased from Sigma-Aldrich, Alfa Aesar, Acros-Organics or J.T. Baker. The free radical initiator 2,2′-azobis(2-methylpropionitrile) (AIBN) was recrystallized from hot toluene prior to use. The co-monomers glycidyl methacrylate (GMA), butyl acrylate (BA) and poly(ethylene glycol methacrylate) (dissolved in isopropyl alcohol, 0.05 g mL^−1^) were passed through a short column filled with inhibitor remover prior to polymerization. The initiator and the co-monomers were stored at −20 °C or +4 °C.

### Size exclusion chromatography (SEC)

SEC measurements were conducted against polystyrene standards in THF at 30 °C. Data were analyzed with the PSS WinGPC Unity software. Sample concentration was 15 mg mL^−1^.

The redox silent polymer matrix poly(glycidyl methacrylate-*co*-butyl acrylate-*co*-poly(ethylene glycol methacrylate)) (P(GMA-BA-PEGMA)) was synthesized following protocols described previously (see Supplementary Fig. 13)^30^. The co-monomers glycidyl methacrylate (GMA, 0.68 mL, 0.710 g, 5 mmol, 49.5 mol%), butyl acrylate (BA, 0.61 mL, 0.545 g, 4.3 mmol) and poly(ethylene glycol methacrylate) (PEGMA, *M*_n = _500 g mol^−1^, 8 mL of a 0.05 g mL^−1^ isopropyl alcohol solution, 0.4 g, 0.8 mmol, 8 mol%) were mixed in a Schlenk tube under argon atmosphere. Then, the free radical initiator AIBN (15 mg, 91 µmol) was added and the mixture was deaerated by bubbling argon through the solution. The reaction mixture was heated to 80 °C and stirred for ≈35 min at this temperature. The turbid solution was quenched with 40 mL of water. The colorless precipitate was separated by means of a centrifuge (4000 rpm, 20 min). The supernatant was decanted off and the residue was successively washed with water (40 mL), MeOH/water (40 mL, 1:1 vol%) and finally with diethyl ether (120 min) with centrifugation and separation after each step. The highly viscous colorless product was dried under reduced pressure. The dry foamy solid was dissolved in acetonitrile (14.5 mL) to obtain a polymer solution with a concentration of 0.1 g mL^−1^ (yield: 1.45 g, 88%). ^1^H-NMR (200.13 MHz, acetone-d_6_) δ/ppm: ≈1 (broad, -CH_3_ and -CH_2_- of backbone); 1.41 (m, -CH_2_-, BA); 1.62 (m, -CH_2_-, BA); 2.69, 2.84 and 3.26 (all s, epoxide, GMA), 3.60 (s, -CH_2_-, PEGMA), 3.82 and 4.37 (all broad, -CH_2_-O moiety, GMA), 4.02 (broad, -CH_2_-O moiety, BA) composition determined via the integral ratio extracted from the ^1^H-NMR spectrum: GMA = 60 mol%, BA = 36 mol%, PEGMA = 4 mol% (Supplementary Fig. 14); SEC (vs. poly(styrene) standard, THF): *M*_w_ = 39 kDa, PDI = 2.8.

### Film formation

Glassy carbon electrodes (GC, Pine Research Instrumentation, 5 mm diameter) were drop-cast with a mixture of 1 mg of CyGly and polymer solution (20 μL, 20 mg ml^−1^ in acetonitrile) and left to dry for 1 h in the glove box. Immobilization of the catalyst on a monolayer was performed as described elsewhere^15^. The pH 3 electrolyte used in all experiments was a 0.1 M MES/0.1 M HEPES mixture where the pH was adjusted with the required amount of HClO_4_.

### Data availability

All data are available from the authors upon reasonable request.

## Electronic supplementary material

Supplementary Information

Peer Review File
