# Supplementary material for: Dual properties of a hydrogen oxidation Ni-catalyst entrapped within a polymer promote self-defense against oxygen
Source: Nat Commun. 2018 Feb 28;9:864. doi: 10.1038/s41467-018-03011-7 (PMC5830441; doi:10.1038/s41467-018-03011-7)
Supplement: Supplementary file 1 — Supplementary Information [file 41467_2018_3011_MOESM1_ESM.pdf]

Supplementary Note 1

The original report emphasized stability of the Ni^{2+} complex to oxygen, matching of the pK_a values of the pendant amino groups to the Ni, and a low ΔG for H_2 addition, which is qualitatively higher for CyGly than for the PhBn[1] complex shown to be fastest in the above mentioned study. The faster rate observed here could be due to higher stability in the presence of O_2, or the matching of the pK_a values of the pendant amines with the Ni. A ^{31}P NMR spectrum measured of the sample used for TOF determination of the O_2 reduction after 2 h exposure to an atmosphere of O_2 showed no oxidized products, suggesting that CyGly is indeed more stable against O_2. ^{31}P NMR was used to analyze the reactivity of the catalyst exposed to an excess of O_2 in the presence of H_2. In addition to the phosphine oxidized product previously reported for a similar complex,[1] several undefined degradation products were obtained.

Supplementary Note 2

Note that the attachment in case of the monolayer to the electrode surface was obtained via the modification of at least one of the ligands within the catalyst, which may reduce its mobility and/or tolerance towards conformational changes that appear during turnover conditions.

Supplementary Methods

^2H NMR experiments for the determination of O_2 oxidation turnover frequency (TOF)

The O_2 reduction to D_2O by CyGly was measured by purging a solution of the catalyst in acetonitrile with D_2 until it turned from purple to yellow (3 minutes) indicating that all of the Ni^{2+}-complex is reduced to the doubly-protonated Ni^0-complex. Then O_2 was bubbled through the solution for five minutes and a ^2H NMR experiment was conducted to quantify the amount of D_2O formed.

The pendant amines of CyGly were deuterated upon the addition of D_2 (Supplementary Figure 5). Note, that the pendant amine-D and the -COOD moieties in the complex can exchange with D_2O formed. Hence, TOFs were calculated under the assumption that the exchange rate of the deuterated N-D and -COOH moieties with D_2O remained constant within the timescale of the experiment.

Products of CyGly degredation upon O_2 exposure

The reactivity of CyGly with O_2 in solution was followed by ^{31}P NMR. CyGly (9.2 mM) was dissolved in CD_3CN, then the NMR tube was flushed with H_2 and a ^{31}P NMR spectrum was measured. Afterwards, the tube was purged with a mixture of $H_2/O_2/N_2$ (90%/5%/5%) for 10 minutes, followed by recording a ^{31}P NMR spectrum. This process was repeated twice, flushing with an $H_2/O_2/N_2$ mixture for 10 minutes prior to each measurement; after the third gas mixture addition, four NMR measurements at 45 minute intervals were conducted (Supplementary Figure 9). After the NMR tube was flushed with H_2, the color of the solution changed from purple to yellow indicating the reduction of CyGly (Ni^{2+} to Ni^0); ^{31}P NMR signals appear in two regions: the signal at -11 ppm, which can be assigned to the exo species, and a signal at around +16 ppm that is assigned to the endo species. After the first addition of O_2, the intensity of the signals at -11 and +16 ppm decreased gradually and a pair of new signals at 0 and +25 ppm as well as several new signals at higher chemical shift appear. The broad signal at +62 ppm is close to the one previously reported for a similar oxidized inactive complex at +66 ppm.[1] The first signal from the starting material to dissapear is the endo species. All initial CyGly signals disapear

almost completely after 175 minutes. To enhance the signal-to-noise ratio, an additional spectra was recorded overnight (Supplementary Figure 10).

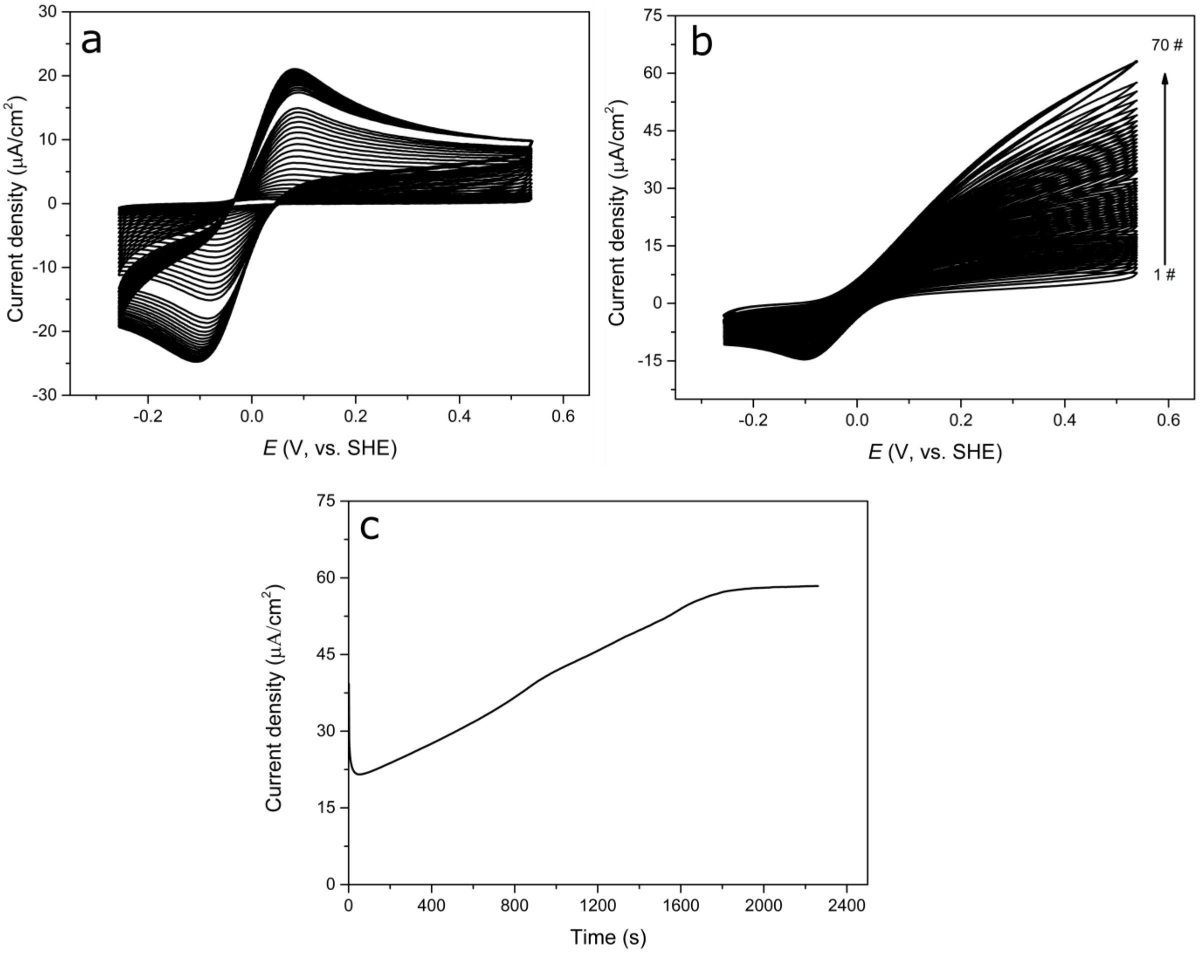

Supplementary Figure 1. Swelling behavior of a CyGly/polymer film followed by means of cyclic voltammetry. The first 40 potential cycles of a freshly prepared CyGly/polymer film at 20 mV s^{-1} under N_2 (**a**) and under H_2 (70 scans, **b**). In (**a**) and (**b**) the last 10 voltammograms did not show any significant change in intensity indicating that swelling reached equilibrium. **(c)**: Chronoamperometry of a freshly prepared CyGly/polymer film at +541 mV vs. SHE. Other conditions: 25°C, pH 3, 2000 rpm.

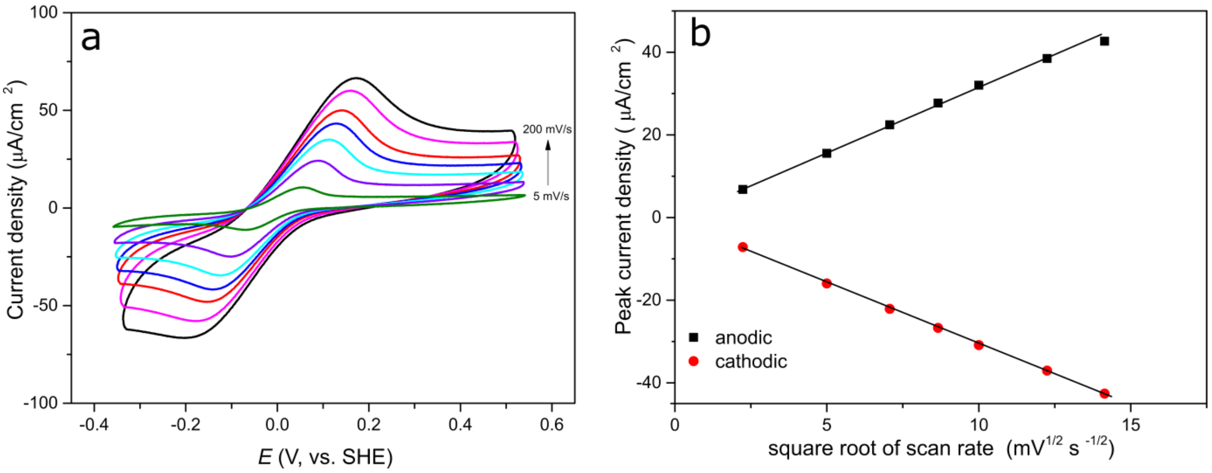

Supplementary Figure 2. (**a**), cyclic voltammograms of a CyGly/polymer film deposited on a GCE measured with different scan rates: 5, 25, 50, 75, 100, 150 and 200 mV s^{-1}. (**b**), anodic (black) and cathodic (red) peak current vs. the square root of the scan rate showing a linear response. Black lines are linear fits over all corresponding data points (R^2_{Adj} is 0.996 and 0.999 for anodic and cathodic currents, respectively). Conditions: pH = 3, 25°C, 2000 rpm under 100% N_2.

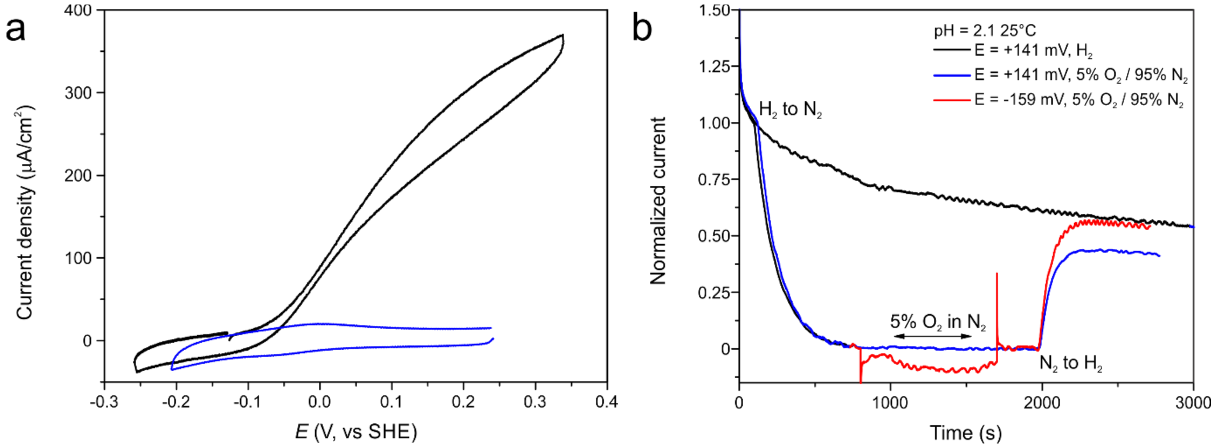

Supplementary Figure 3. (a) Cyclic voltammograms of a CyGly monolayer under N_2 (blue trace) and under H_2 (black trace) recorded with a scan rate of 20 mV s^{-1}. **(b)** Chronoamperometric experiments of a CyGly monolayer. H_2 was first replaced by N_2 then 5% O_2 was added over 10 min (indicated by an arrow) afterwards O_2 was removed and H_2 was added back to the gas flow; in the blue and black trace, the potential was set to +141 mV vs. SHE throughout the experiment while in the red trace it was switched to -159 mV vs. SHE during O_2 addition then switched back to +141 mV vs. SHE. The black trace is a control, showing the usual decay of the catalytic activity at +141 mV vs. SHE under H_2. Conditions: 25°C, 2000 rpm, pH 2.1 ($HClO_4$ 0.1M), currents in (**b**) were normalized to the current value when switching the gas flow from H_2 to N_2 at t = 300 s.

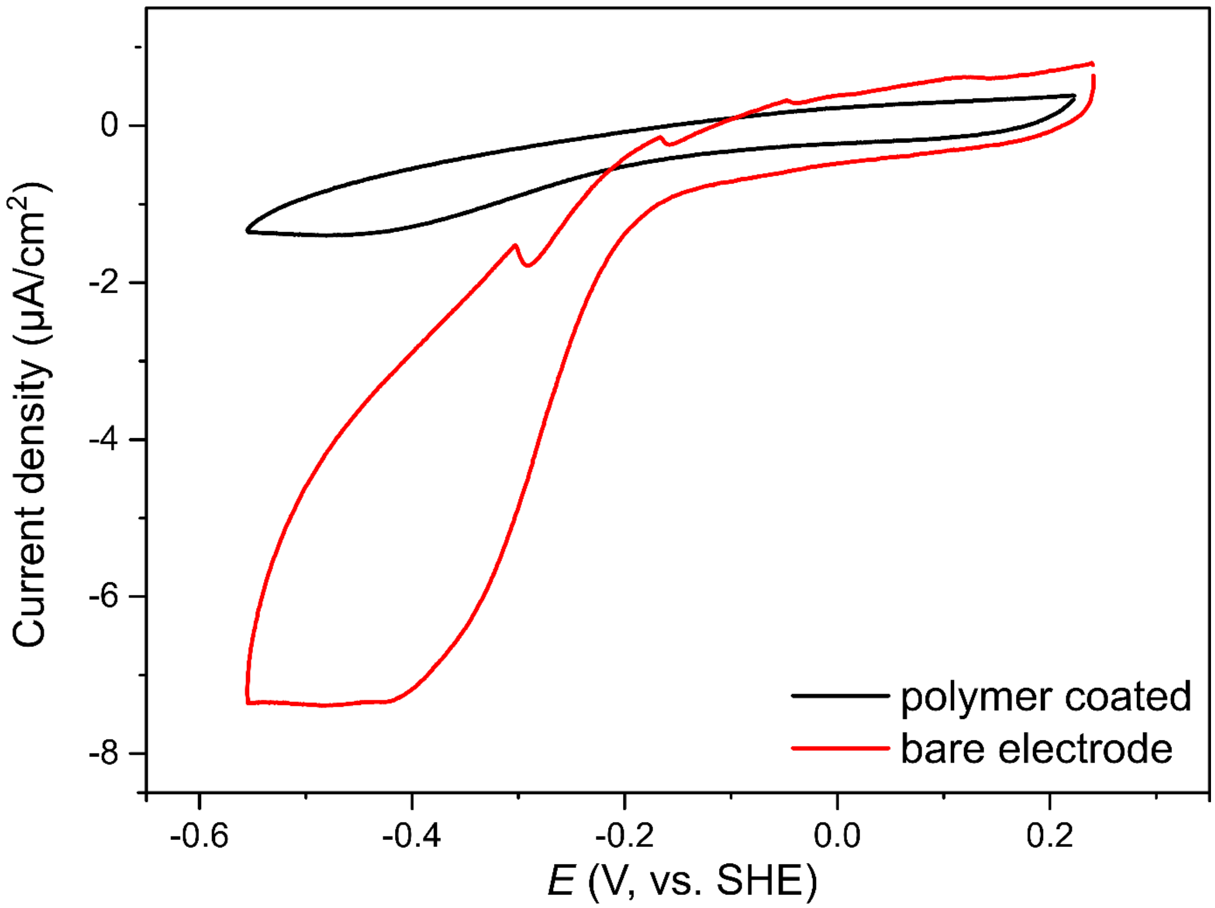

Supplementary Figure 4. O_2 reduction at the GCE surface. Cyclic voltammograms of a bare glassy carbon electrode under O_2 (red trace) and a polymer-coated electrode (black trace). Conditions: 20 mV s^{-1}, 25°C, pH 3.

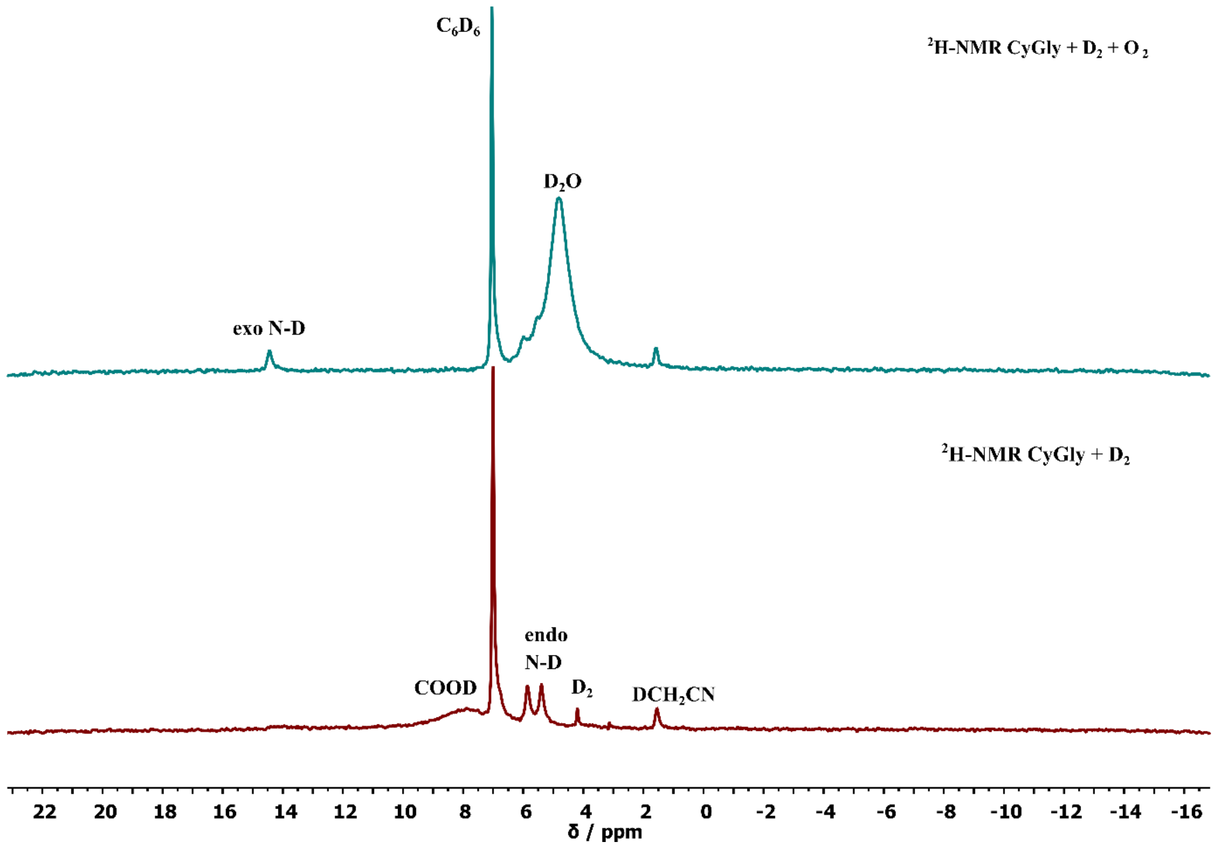

Supplementary Figure 5. ^{2}H NMR (76.7 MHz) spectra of CyGly in CD_3CN bubbled with D_2 for 3 minutes (red trace) and after bubbling 5 minutes with O_2 (blue trace).

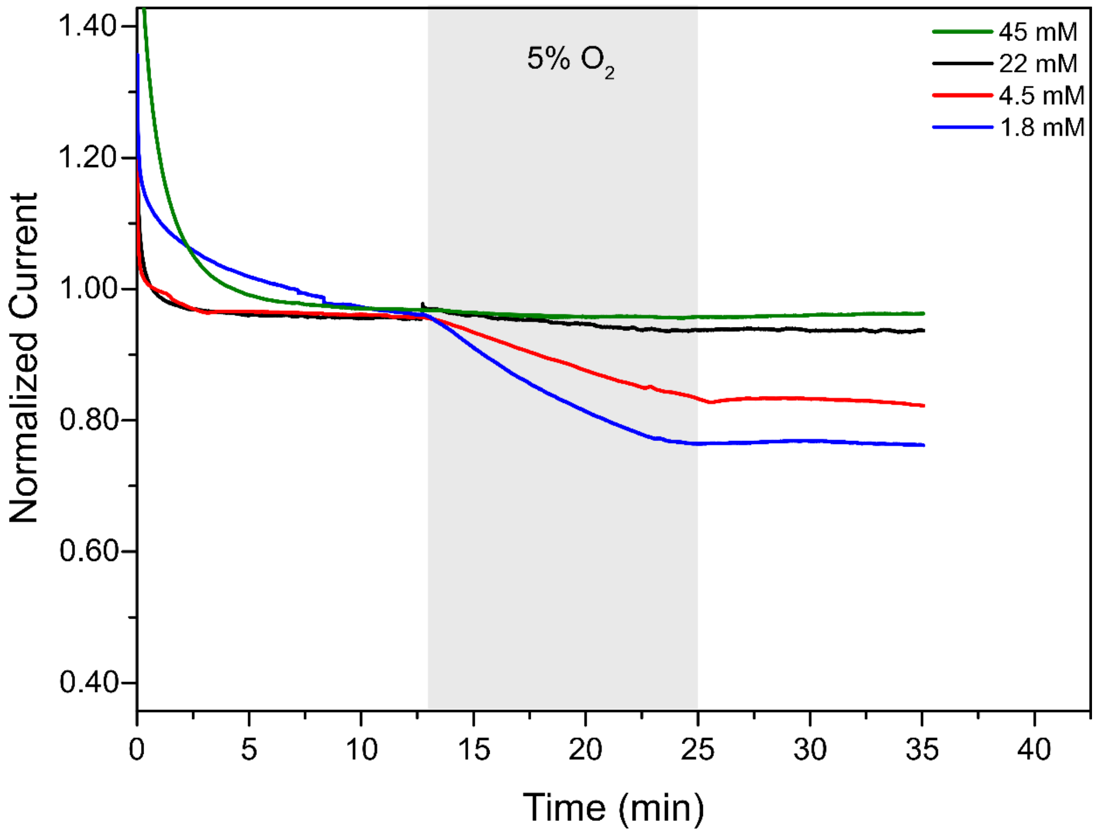

Supplementary Figure 6. Comparison between chronoamperometry experiments of CyGly/polymer films with different catalyst loadings by changing its concentration (see graph) in the drop-cast solution at a constant polymer concentration (give value). Exposure to O_2 for 10 min is indicated with the shadowed area. Conditions: +541 mV vs. SHE, pH 3, 25°C, 2000 rpm. Currents were normalized to the point at which O_2 was added to the gas feed, *t* = 13 min.

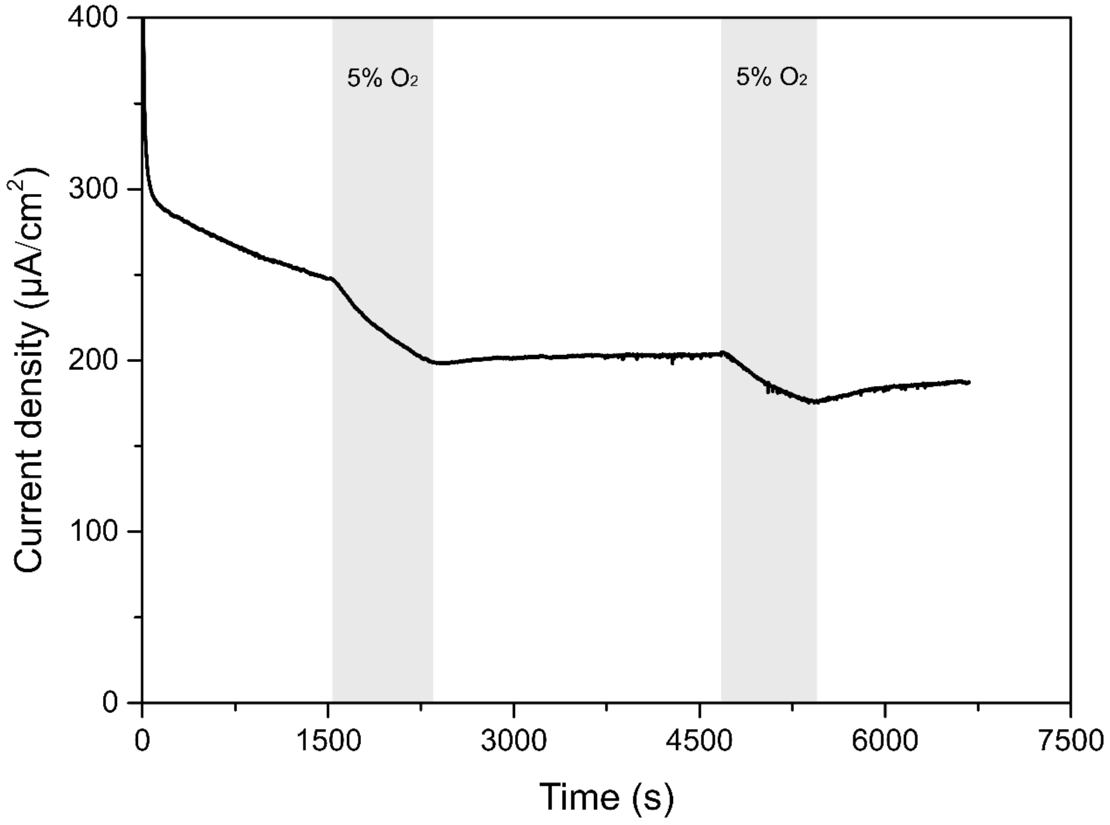

Supplementary Figure 7. Chronoamperometry of a CyGly/polymer/CNT film deposited on a GCE under 90 % H_2 in N_2 with addition of O_2 as indicated. The conductive CNT's increase the catalytic current significantly, but also increase the sensitivity to oxygen. Conditions: +541 mV vs. SHE, 25°C, pH = 3, 2000 rpm.

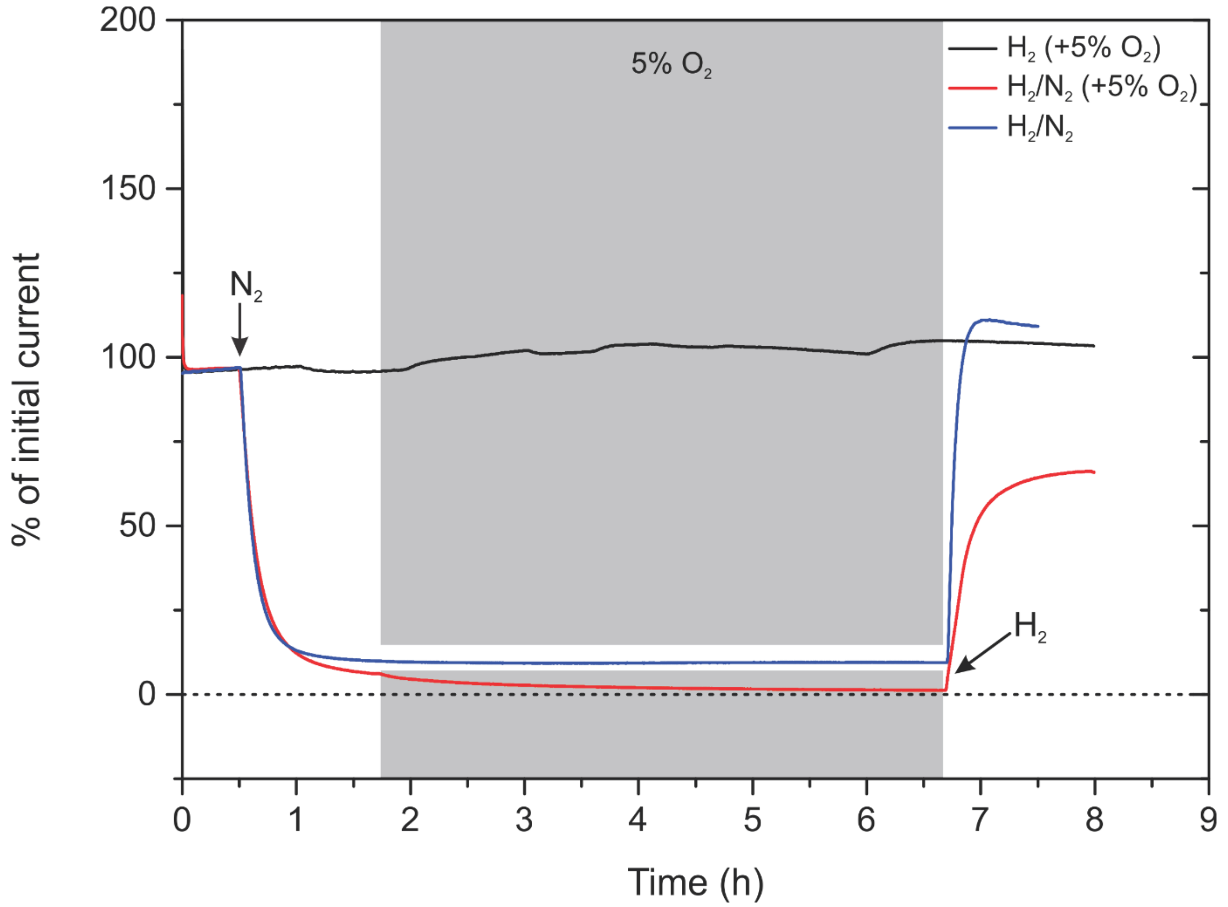

Supplementary Figure 8. Chronoamperometry experiments of three CyGly/polymer films deposited on individual GCEs. The initial gas mixture is 90 % H_2 in N_2 for all three experiments to evaluate the catalytic current before O_2 exposure. The currents were normalized to this value. The black trace corresponds to an electrode that was exposed to a 5% O_2, 90% H_2 and 5% N_2 mixture for 5 h. For the red and blue traces, after 30 min. the gas was switched to 100% N_2 and for the red trace a 5% O_2 was added to the gas mixture for 5 h. On the final step, the gas flow was switched back to the initial 90% H_2 in N_2 composition. Conditions: +541 mV vs. SHE, 25°C, pH = 3, 1000 rpm.

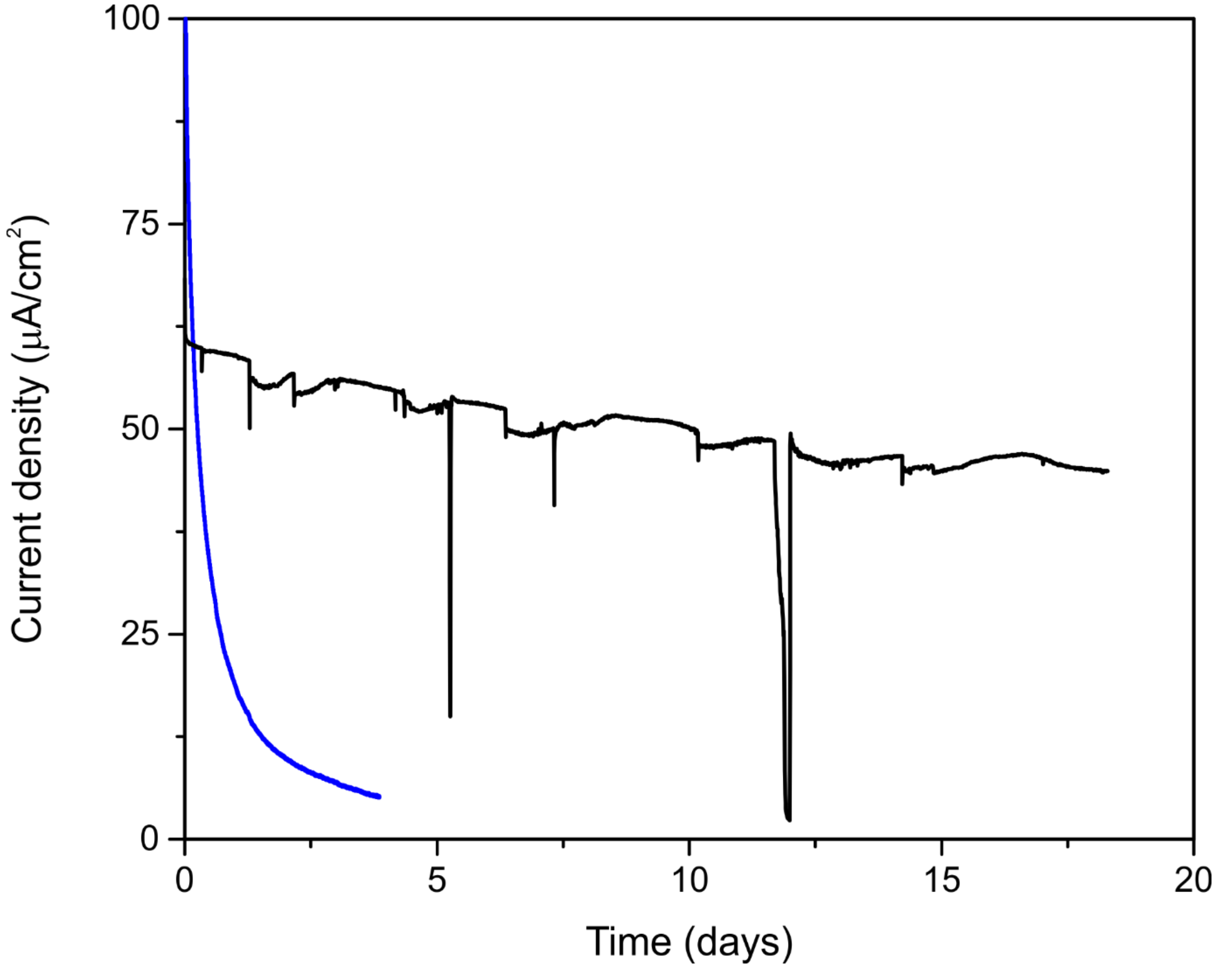

Supplementary Figure 9. Long term stability test (18 days) of a CyGly/polymer film under continuous turnover conditions evaluated by means of chronoamperometry (black trace). Conditions: 100% H_2 (250 mL min^{-1}), applied potential: +541 mV vs. SHE, 25°C, pH = 3, 500 rpm. H_2 flow and electrode rotation rate were lowered to avoid excessive gas use and rotator degradation. As a result, currents are lower than elsewhere in the manuscript. Current fluctuations are due to cell maintenance like buffer addition and gas bottle change. For comparative purposes, the chronoamperometric data of a CyGly monolayer (data obtained from[2]) is also shown (blue trace).

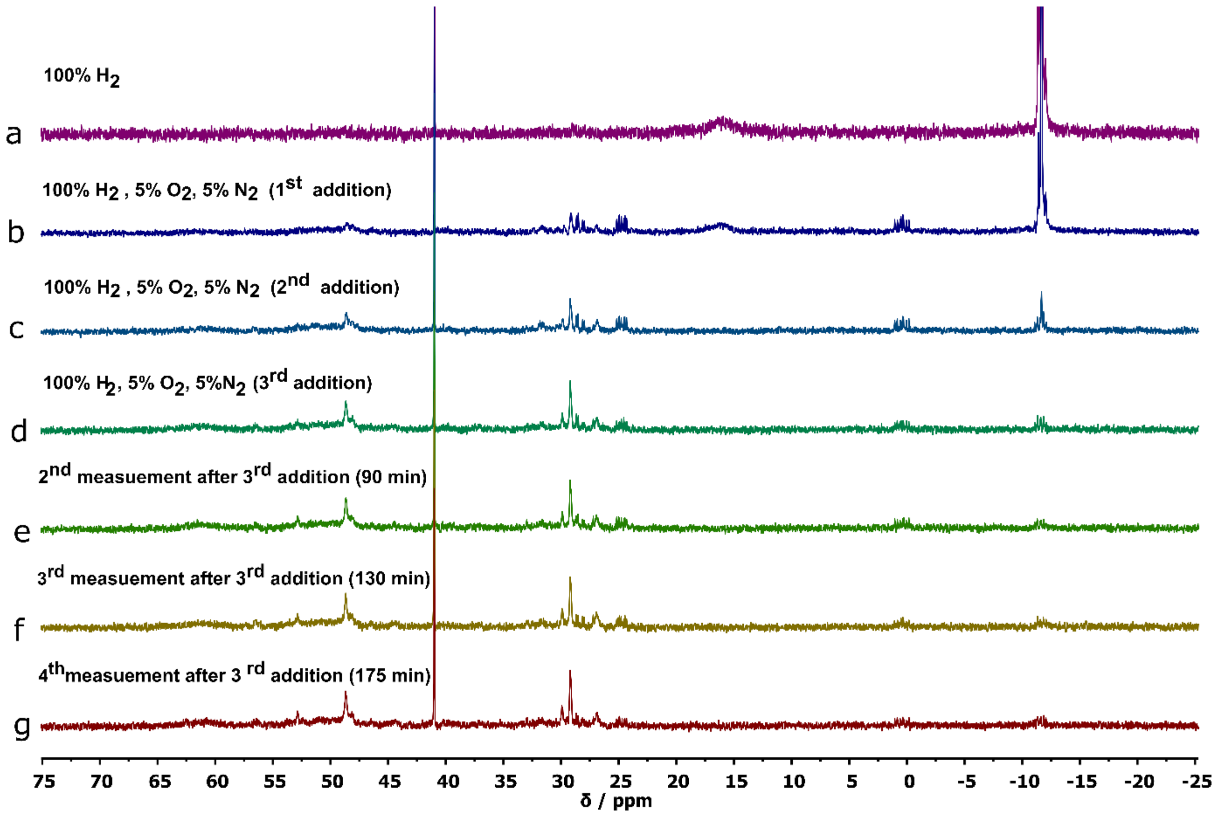

Supplementary Figure 10. ^{31}P NMR (202.4 MHz) spectra of CyGly in CD_3CN. First spectrum (a) was recorded under 100 % H_2 then the tube was flushed with $H_2/O_2/N_2$ (90 %/5 %/5 %) gas mixture and the second spectrum (b) was measured. Two additional experiments (c, d) were conducted for which the NMR tube was flushed with the same gas mixture prior to each measurement. After the third gas mixture addition, four spectra (d-g) were recorded at 45 min intervals.

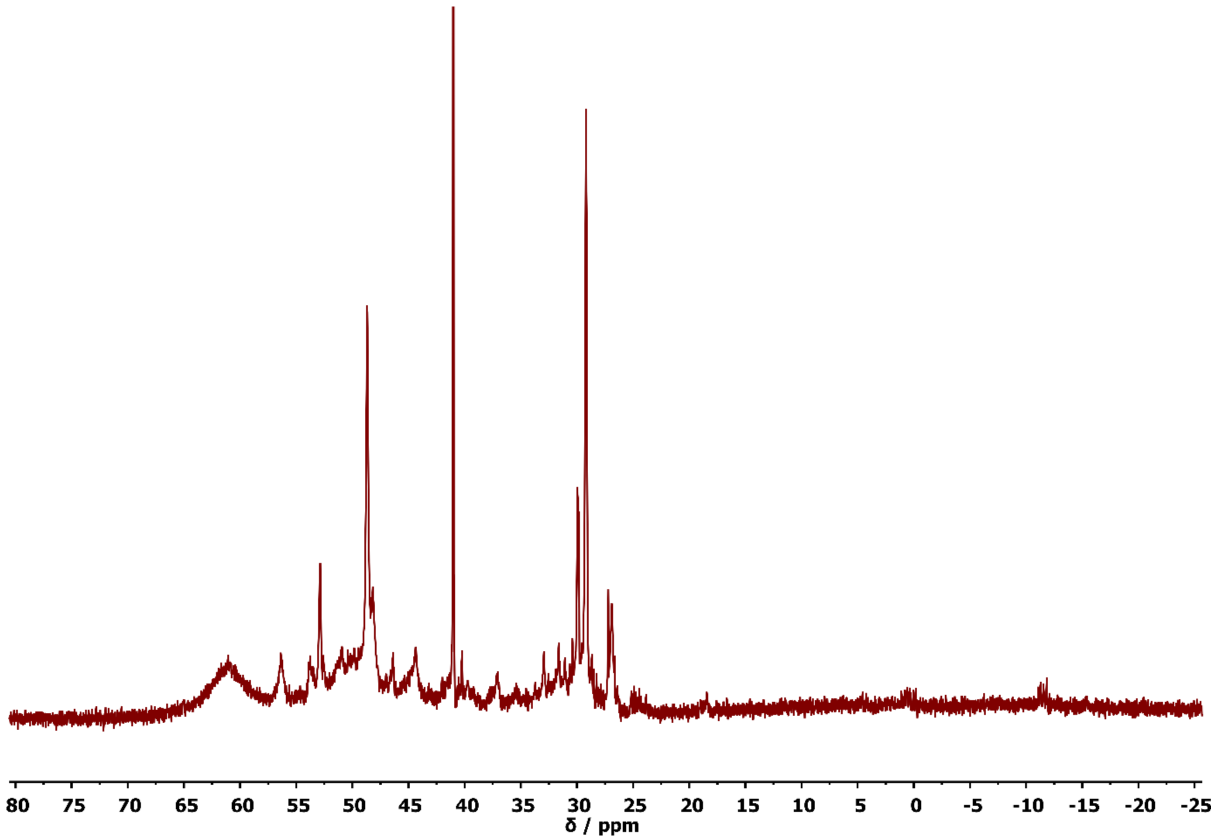

Supplementary Figure 11. ^{31}P NMR (202.4 MHz) spectrum of CyGly in CD_3CN after flushing the NMR tube with $H_2/O_2/N_2$ (90%/5%/5%) gas mixture 3 times. Signals of the parent CyGly complex had almost completely disappeared. The spectrum was obtained from averaging 20500 scans overnight.

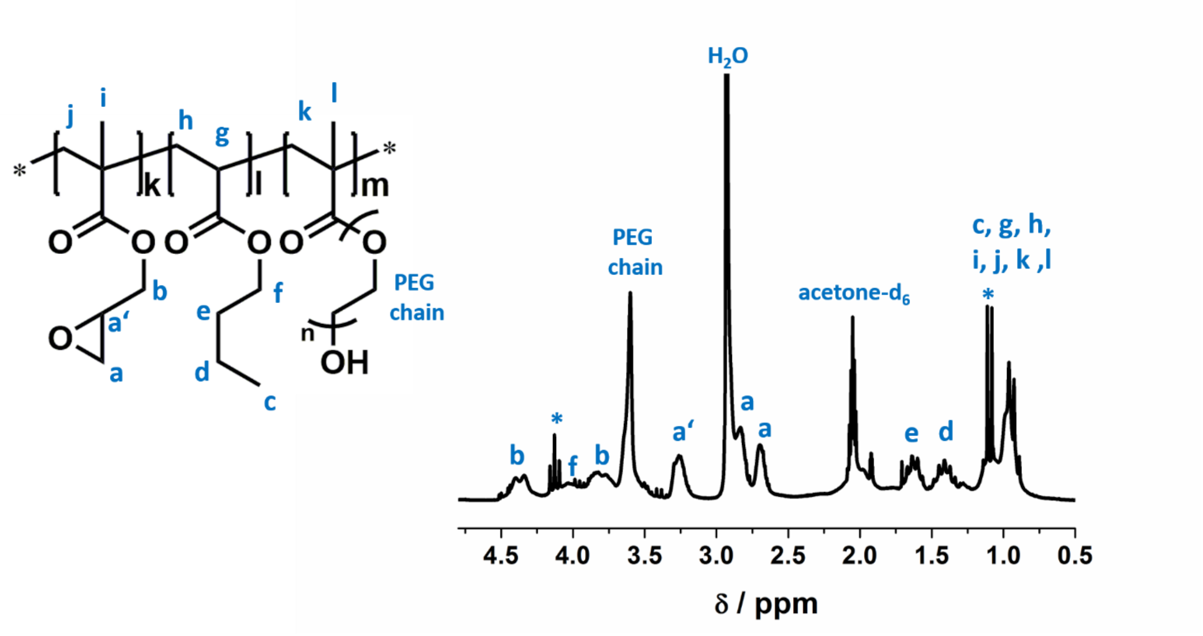

Supplementary Figure 12. ^{1}H-NMR spectra (200.13 MHz, acetone-d_6) and molecular structure of the polymer P(GMA-BA-PEGMA) including signal assignment; the residual solvent peak (acetone-d_6) was used as internal standard; k = 60 mol% , l = 36 mol% , m = 4 mol%; the composition of the polymer backbone was calculated from the integral ratios of signals a´ (GMA), d (BA) and the signal of the PEG chain (PEGMA).

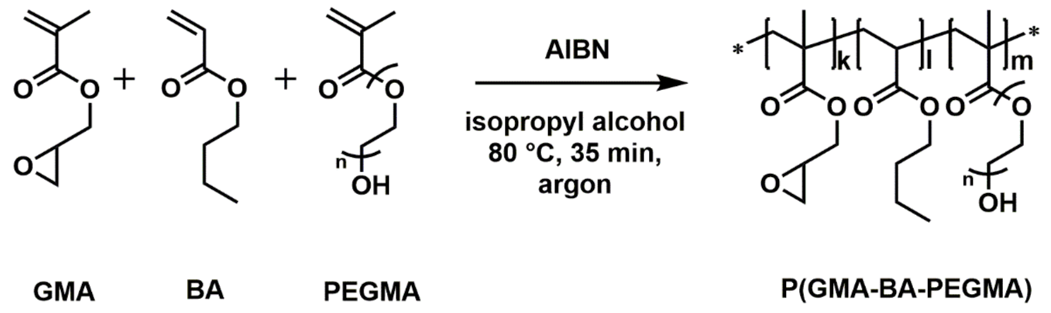

Supplementary figure 13. Synthesis of the redox silent polymer matrix. P(GMA-BA-PEGMA) is synthesized via a free radical polymerization reaction initiated by the thermal decomposition of AIBN. Nominal composition: k = 49.5 mol%, l = 42.5 mol%, m = 8 mol %; actual composition (determined via NMR): k = 60 mol%, l = 36 mol%, m = 4 mol %.

Supplementary References

1 Yang, J. Y. *et al.* Reduction of oxygen catalyzed by nickel diphosphine complexes with positioned pendant amines. *Dalton Trans.* ***39***, 3001-3010 (2010).

2 Rodriguez-Macia, P.; Dutta, A.; Lubitz, W.; Shaw, W. J.; Rüdiger, O., Direct comparison of the performance of a bio-inspired synthetic nickel catalyst and a [NiFe]-hydrogenase, both covalently attached to electrodes. *Angew. Chem. Int. Ed. Engl.* ***54***, 12303-7. (2015)
